# Sea Surface Temperature Modulates Physiological and Immunological Condition of *Octopus maya*

**DOI:** 10.3389/fphys.2019.00739

**Published:** 2019-06-25

**Authors:** Cristina Pascual, Maite Mascaro, Rossanna Rodríguez-Canul, Pedro Gallardo, Ariadna Arteaga Sánchez, Carlos Rosas, Honorio Cruz-López

**Affiliations:** ^1^Unidad Multidisciplinaria de Docencia e Investigación, Facultad de Ciencias, Universidad Nacional Autónoma de México, Sisal, Mexico; ^2^Laboratorio Nacional de Resiliencia Costera, Consejo Nacional de Ciencia y Tecnología, Sisal, Mexico; ^3^Laboratorio de Inmunología y Biología Molecular, Centro de Investigación y de Estudios Avanzados del Instituto Politécnico Nacional, Unidad Mérida, Merida, Mexico; ^4^Posgrado en Ecología Molecular y Biotecnología, Universidad Autónoma de Baja California, Ensenada, Mexico

**Keywords:** *Octopus maya*, hemolymph, eco-immunology, physiology, immunological indicators

## Abstract

*Octopus maya* is a valuable endemic species of the Yucatán Peninsula (YP). This area can be divided into distinct regions depending on the presence of cold waters associated to upwelling events during spring and summer. This study was designed to determine if the physiological and immunological condition of *O. maya* show a relationship with variation of the sea surface temperature associated with the seasonal upwelling. A total of 117 organisms were collected from February to July in three fishing zones: Ría Lagartos located in the upwelling zone; Seybaplaya corresponding to the non-upwelling zone, and Sisal, the transitional zone. The organisms were examined in terms of physiological (total weight, the weight of the gonad and digestive gland, osmotic pressure, hemocyanin, protein, glucose, and cholesterol concentrations in plasma), and immunological variables (total hemocyte count, hemagglutination, phenoloxidase system activity, total phenoloxidase plasma activity, and lysozyme activity). Multivariate one-way ANOVA showed overall significant differences between groups of octopus by month/zone of capture, indicating that the physiological-immunological condition of *O. maya* is related to a temperature gradient. Wild octopuses captured at the upwelling zone and the transitional zone (Ría Lagartos and Sisal) in February, March, and April -with temperatures lower than 27°C- were in better conditions: larger size, high concentrations of hemocyanin, and low activity of the phenoloxidase system. Octopuses captured in the warmer waters (28–30°C) of the non-upwelling and transitional zones (Seybaplaya and Sisal) during June and July, could be reflecting the metabolic stress through immunological compensation mechanisms with higher activity of the phenoloxidase system, despite having a lower concentration of hemocytes, hemocyanin, and proteins. Although the movement of individual *O. maya* along the YP throughout their life cycle has not yet been determined, direct development and benthic behavior could limit the mobility of the organisms in such a way that their physiological and immunological condition might reflect adaptation to the regional environment. This information could help understand the performance of octopuses in their distribution area, which sustains an important fishery.

## Introduction

*Octopus maya* (Voss and Solís-Ramírez, 1966) is an endemic species of the Yucatán Peninsula (YP), Mexico. This species alone sustains the main *Octopus* fishery in the American continent with annual production ranging from 8,000 to 20,000 tons (Markaida et al., 2017). The geographical distribution of *O. maya* is the smallest in size among all the cephalopod’s species, and it coincides with the area of influence of summer upwelling of sub-superficial subtropical waters from de Caribbean to the YP shelf characterized by temperatures between 16 and 22°C (Enriquez et al., 2013). This upwelling affects only the eastern portion of the YP continental shelf, resulting in a summer eastern-to-western thermal gradient with low-to-high temperatures, offering different and unique environments to aquatic species of the zone (Zavala-Hidalgo et al., 2006). The life cycle of *O. maya* occurs in the YP and is of around 8–12 months (Hanlon and Forsythe, 1985). Females of the species may lay from 1,500 to 2,000 eggs with average size of 17 mm in length and produce large benthic hatchlings measuring around 6–7 mm mantle length (Roper et al., 1984; Caamal-Monsreal et al., 2015; Tercero et al., 2015).

*Octopus maya* is an ectothermic organism, and recently relevant knowledge has been gained to understand its thermal tolerance in experimental studies. When octopus are exposed to high temperatures (>28°C) adverse effects are observed on growth (Noyola et al., 2013a, b), reproductive efficiency (Caamal-Monsreal et al., 2016; López-Galindo et al., 2018), and embryonic development (Juárez et al., 2015). However, little is known about the effect of changes on temperature on the physiology and immune response of *O. maya*. In the face of the climatic change scenario, there is relevant information showing a greater susceptibility to opportunistic infections at the population level (Paillard et al., 2004; Mydlarz et al., 2006). The main results of ocean warming are the decrease of oxygen levels and acidification in the coastal waters to mention some. Hypoxia can directly affect the immunocompetence of marine invertebrates, but the mechanical links between temperature and immune response are still unsolved (Mydlarz et al., 2006). In this sense, the biochemical and immune metabolites measured in the hemolymph can provide information to assess the health status and physiology of a given organism (Fazio, 2019). This approach has been widely used for clinical diagnosis in veterinary settings.

The immune system is involved in the preservation of the biological integrity of living organisms as it allows the recognition and neutralization of non-self-molecules either from the environment or produced by metabolic processes. The defense mechanisms of invertebrates are considered simple since they lack processes that are present in vertebrates, such as immunoglobulin-mediated immune memory. Nevertheless, modern cephalopods appeared at the same time as bony fish, more than 200 million years ago (Hochner et al., 2006), indicating they have developed similarly diverse, successful strategies to cope with infections and other harmful elements of the environment.

So far, in cephalopods, the main line of defense from foreign substance rely on peripheral cells or hemocytes which have phagocytic, encapsulation and neutralization capabilities and are also involved in inflammatory and injury healing processes (Beuerlein et al., 2002). In invertebrates the amplification of defense mechanisms is associated to the prophenoloxidase (proPO) system found within hemocytes granules (Söderhäll and Smith, 1983; Söderhäll and Häll, 1984). The proPO system is directly released when hemocytes are stimulated by fungal or bacterial beta-glucans (βG) or lipopolysaccharides (LPS) (Söderhäll and Häll, 1984); signals associated with the wound and cellular damage (DAMPs) (Dubovskiy et al., 2016), or by recognition serum proteins which warn hemocytes (Vargas-Albores et al., 1996, 1997). Once activated, the proPO system produces several factors that stimulate hemocytes to eliminate the foreign material by phagocytosis, nodule formation and/or encapsulation (Söderhäll and Smith, 1983; Sung et al., 1998). Phagocytosis is one of the most important mechanisms of hemocytes immune response. During this process, phagolysosomes are formed, and highly reactive lytic substances such as peroxide, superoxide, and nitric oxide derivatives are released (Muñoz et al., 2000; Campa-Córdova et al., 2002). The process is known as a respiratory burst and has an essential role in hemocytes microbicide activity (Song and Hsieh, 1994).

Among components of the immune system are lectins, a very diverse group of proteins capable of recognizing carbohydrates. Several studies have pointed out the capacity of *O. vulgaris* plasma to agglutinate bacteria and erythrocytes (Rögener et al., 1987). Fisher and DiNuzzo (1991) conducted a comprehensive study that demonstrated hemagglutination activity against isolated bacteria and seven types of erythrocytes of octopus *O. maya*, Japanese squid, *Sepioteuthis lessoniana*; and common cuttlefish, *Sepia officinalis*. These results demonstrated the diverse recognition abilities and the existence of molecules that participate in the hemagglutination processes. Lysozymes —a group of proteases capable of hydrolyzing components of microorganism’s surface —, are also found among the humoral effectors of the immune response. These enzymes are found in hemocytes vacuoles and are secreted to the hemolymph by these and other cells (Locatello et al., 2013).

Cephalopods have a closed circulatory system which transports hemolymph through blood vessels and capillaries. Plasma metabolites concentrations reflect metabolic adjustments associated to the type of food ingested, energy demands and physiological adaptations that take place when organisms are exposed to different environmental conditions (Pascual et al., 2003, 2004). The main evaluation criteria used to determine the physiological condition of cephalopods at population level include biochemical characterization of the digestive gland, gonads, and muscle tissues (Rosas et al., 2002; Sieiro et al., 2006; Gallardo et al., 2017). The mobilization of reserves has been associated with the weight of the digestive gland, one of the main catabolism organs. In previous studies, we determined that the plasmatic metabolites of *O. maya* (total proteins, acylglycerols, cholesterol, and glucose), are related to the quality of the dietary intake as well as their nutritional condition (Aguila et al., 2007; Moguel et al., 2010; Martínez et al., 2014; Linares et al., 2015). In marine invertebrates the metabolites variation has been related to the general physiological condition that helps to understand what kind of metabolic route is used under a given condition; acclimation temperature (Sánchez et al., 2001); size-based selection program on blood metabolites and immune response (Pascual et al., 2004); and immune response against a specific viral infection (Pascual-Jiménez et al., 2012).

This study was developed to evaluate if the variations on the sea surface temperature related to the seasonal upwelling of the Yucatán Peninsula, Mexico, had a relationship with the health status of the octopuses. Based on the information on the thermal tolerance of the species, we hypothesized that the octopuses captured in the localities and months with sea surface temperatures >28°C will present a less optimal physio-immunological condition than the organisms captured in localities and months with lower surface temperature (23–26°C). This information would help to understand the physiological–immunological performance of octopuses in its distribution area that sustains an important fishery activity.

## Materials and Methods

### Sampling Locations

The Campeche Bank is located on the continental shelf of the YP adjacent to the states of Yucatán and Campeche in southwestern Gulf of Mexico. Oceanographic conditions on the shelf, and the presence of local upwelling allows differentiation of three environmental zones with contrasting temperature regimes (Enriquez et al., 2013). Samples of octopus *O. maya* were collected at three locations from February to July, 2010: Ría Lagartos (21°38′N, 88°10′W) located in the upwelling zone (Z1); Seybaplaya (19°38′N, 90°41′W) corresponding to a zone with no influence of upwelling (Z3); and Sisal (21°09′N, 90°01′O), a zone of transition located between the two former locations (Z2). The main upwelling event occurs from July to September in Z1, is less prominent in Z2, and is absent in Z3 (Merino, 1997).

### Octopus Sampling

A total of 117 organisms were analyzed: 24 from Ría Lagartos, 66 from Sisal, and 27 from Seybaplaya. The total weight of the captured organisms ranged from 100.67 to 1,934.2 g Immediately after capture, octopuses were placed in a closed tank with seawater that was kept circulating using a submersible water pump. The boat headed to the dock where they were transferred to a collecting tank connected to a 250 L tank used for transportation to laboratory facilities at UMDI-Sisal, Yucatán, Mexico. Total transportation from the dock to the laboratory lasted around 2–7 h, and the temperature of the water during transportation fluctuated between 25 and 28°C. Each transportation tank was provided with several 4-inch PVC tubes that were placed inside as individual shelters to reduce interaction stress among the organisms. Once in the laboratory, organisms were placed individually in 80 L tanks with seawater at 27–28°C with constant flow and aeration. Water passed through 5 μm filters with replacement rate equivalent to 300% per day. In such conditions, it was possible to maintain ammoniacal nitrogen and nitrite levels below 0.1 mg ml^-1^, nitrate below 50 mg ml^-1^, and pH between 7.7 and 8.2, values recommended as appropriate to keep different species of octopus in captivity, including *O. maya* (Hanlon and Forsythe, 1985).

### Hemolymph Sampling

Before hemolymph sampling, octopuses were anesthetized by hypothermia at 10°C for several minutes (Cruz-López, 2010; Linares et al., 2015; Roumbedakis et al., 2017). This was observed physically by reduction of the breathing rate (indicated by contractions of the mantle), and reduction of locomotor activity. After that, each animal was removed from the cold water and hemolymph was drawn from the cephalic aorta using a pre-chilled catheter connected to a 5 ml Falcon tube and immediately kept refrigerated (2–8°C) to be used few hours later (Cruz-López, 2010). Hemolymph was centrifuged at 800 × *g* for 5 min at 4°C to separate the plasma, which was used to evaluate plasmatic metabolites, phenoloxidase (PO) and hemagglutination activity. The cellular pellet from each sample was washed twice with isotonic solution (IS: 0.45 M NaCl, 10 mM KCl, 10 mM HEPES, 7.3 pH, and 10 mM EDTA–Na_2_) and centrifuged as described above. The cellular pellet was then re-suspended several times with cacodylate buffer (10 mM cacodylic acid, 10 mM CaCl, pH 7.0) in an equal volume of hemolymph and centrifuged at 13,000 × *g* for 5 min at 4°C. The supernatant was used to evaluate the PO activity from degranulated hemocytes.

### Physiological Variables

Soon after hemolymph collection, octopuses were euthanized by brain puncture (Boyle, 1976; Fiorito et al., 2015). Thereafter, animals were weighed (total weight), and the gonad and the digestive gland were removed and weighed separately. Glucose, cholesterol, and acylglycerides concentrations in plasma were determined in triplicate in 96-well flat bottom plates using specific commercial chromogenic kits (Sera Pack Plus Bayer^®^), adding 10 μl of plasma to 200 μl of the appropriate enzyme reagent for each sample. Protein concentrations were also determined in triplicate in 96-well flat bottom plates. The plasma was previously diluted in sterile water (400×) and then 10 μl of this solution was mixed with 200 μl of commercial solution (Biorad Protein assay 500-0006) according to the Bradford (1976) method. Bovine serum albumin was used as a standard. Absorbance values of all metabolites were recorded in a microplate reader (Benchmark Plus BioRad). A standard curve was developed for each metabolite and linearity was confirmed. The concentration (mg ml^–1^) of samples were calculated using the standard curves.

Hemocyanin concentration was measured by placing 10 μl of hemolymph diluted in 990 μl of Tris 0.1M, pH 8.0, in a 10-mm cuvette. The absorbance was measured at 335 nm (Genesys 10 UV-Vis, Thermo Scientific). Hemocyanin concentration was calculated using an extinction coefficient of 17.26 calculated on the basis of the functional subunit of 74 kDa (Chen and Cheng, 1993a, b). To measure the osmotic pressure, we placed 20 μl of hemolymph in a micro-osmometer (3MO Plus, Advanced Micro-osmometer). Results were expressed as mOsm kg^-1^ (Lignot et al., 2000).

### Immunological Variables

Hemagglutination activity was measured using human blood (type O+) obtained from a local blood bank. Samples of 50 μl of octopus plasma were added to a U-shaped 96-well microliter plate, and twofold serial dilution were prepared using 0.9% saline solution as the diluent. Prior to use, erythrocytes were washed three times with 0.9% saline solution, centrifuged at 380 × *g* at 25°C for 5 min, and then adjusted to a final volume of 2%. An equal volume of the erythrocyte solution was added to each well and incubated for 3 h at room temperature. In controls, plasma was replaced by 0.9% saline solution. Plasma hemagglutination titer was expressed as the reciprocal of the highest dilution showing a positive visible pattern of agglutination (Pascual-Jiménez et al., 2012).

Phenoloxidase system activity was measured by spectrophotometry in triplicate in 96-well flat bottom plates (Hernández-López et al., 1996). The technique was adjusted for *O. maya* (Roumbedakis et al., 2017). The plasma and degranulated hemocytes plasma of 50 μl samples were incubated for 10 min at 37°C to transform proPO into phenoloxidase (PO) without using exogenous trypsin. To evaluate total phenoloxidase activity, plasma was incubated with 50 μl of trypsin (bovine pancreatic 0.1 mg ml^–1^; Sigma T8003). Then, 180 μl of L-3,4-dihydroxyphenylalanine (L-DOPA, 3 mg ml^–1^; Sigma D9628) were added to each well and the microplate incubated for more than 10 min at 37°C. Absorbance was measured at 490 nm in a microplate reader (Benchmark Plus BioRad). Results were expressed as the increment of 0.001 in optical density.

Total hemocytes were counted in a Neubauer chamber from a hemolymph aliquot fixed with 4% formaldehyde in Alsever solution (115 mM C_6_H_12_O_6_, 30 mM Na_3_C_6_H_5_O_7_, 338 mM NaCl, 10 mM EDTA.Na_2_, pH 7.0) with a 1:3 dilution (Roumbedakis et al., 2017). Samples were kept at 2–8°C, for a maximum period of 10 days before analysis. Counting was performed in duplicate covering a minimum area count of 0.04 mm^–3^ and expressed as cells mm^–3^.

Lysozyme activity was quantified according to the turbidimetric method of Parry et al. (1965) with slight modification. *Micrococcus* were suspended in 0.05 M sodium phosphate buffer (pH 7.3), transferred to a cuvette and read at UV-vis spectrophotometer at 530 nm. Hemolymph (100 μl) was transferred to the cuvette, and the reduction in absorbance was recorded. The result was expressed as U ml^–1^.

To avoid immune system activation by endotoxins, all glassware was washed with Etoxa-clean prior to use and solutions were prepared using pyrogen-free water and filtered through a 0.2 μm Acrodisc.

### Statistical Analysis

Multivariate analyses are useful to determine whether individuals can be characterized by a set of attributes (i.e., physiological and immunological variables). With a principal coordinate analysis (PCoA, Legendre and Legendre, 1998), we used a set of 13 physiological and immunological variables to order the organisms. Localities analyzed are close to the limits of the geographic distribution of *O. maya* (west, east, and intermediate zones), and reflect, in turn, the influence of seasonal upwelling and the pattern of sea surface temperature on the coast of the Yucatán Peninsula.

Physiological descriptors were total weight (Wtot g), digestive gland (Wdgl g) and gonad weight (Wgon g), glucose (Glucose mg ml^–1^), cholesterol (Cholest mg ml^–1^), plasmatic proteins (Protein mg ml^–1^), osmotic pressure (OP mOml kg^–1^) and hemocyanin concentration (Hemocy mM). Immunological descriptors were total hemocyte count (THC cells mm^–3^), hemagglutination activity (Hemag titer), lysozyme activity (Lyzoz U ml^–1^), phenoloxidase system activity (PO Sys OD 490 nm) and total phenoloxidase activity in plasma (PO Plas OD 490 nm). Samples (*n* = 117) were analyzed using Gower’s dissimilarity index (Legendre and Legendre, 1998). Weight data was previously square root transformed. A permutational MANOVA was used to examine variations in these descriptors amongst octopus combining month and zones of collection (Anderson, 2001) (see the section “Sampling Locations”). The underlying experimental design was a one-way model with six levels (February, March, and June from the transitional zone at Sisal; March and June from the upwelling zone at Ría Lagartos; and July from the non-upwelling zone: Seybaplaya). Because the number of samples differed among levels, a Type III sum of squares was used for the partitioning of total variation. A maximum of 9,999 unrestricted permutations of raw data were used to obtain the empirical distribution of *pseudo-F* values (Anderson, 2001; McArdle and Anderson, 2001). Multivariate paired comparisons between all six centroids were obtained following a similar procedure to calculate empirical *pseudo-t* values. In addition, two tables with values of physiological and immunological descriptors was make by locality.

## Results

**FIGURE 1:**
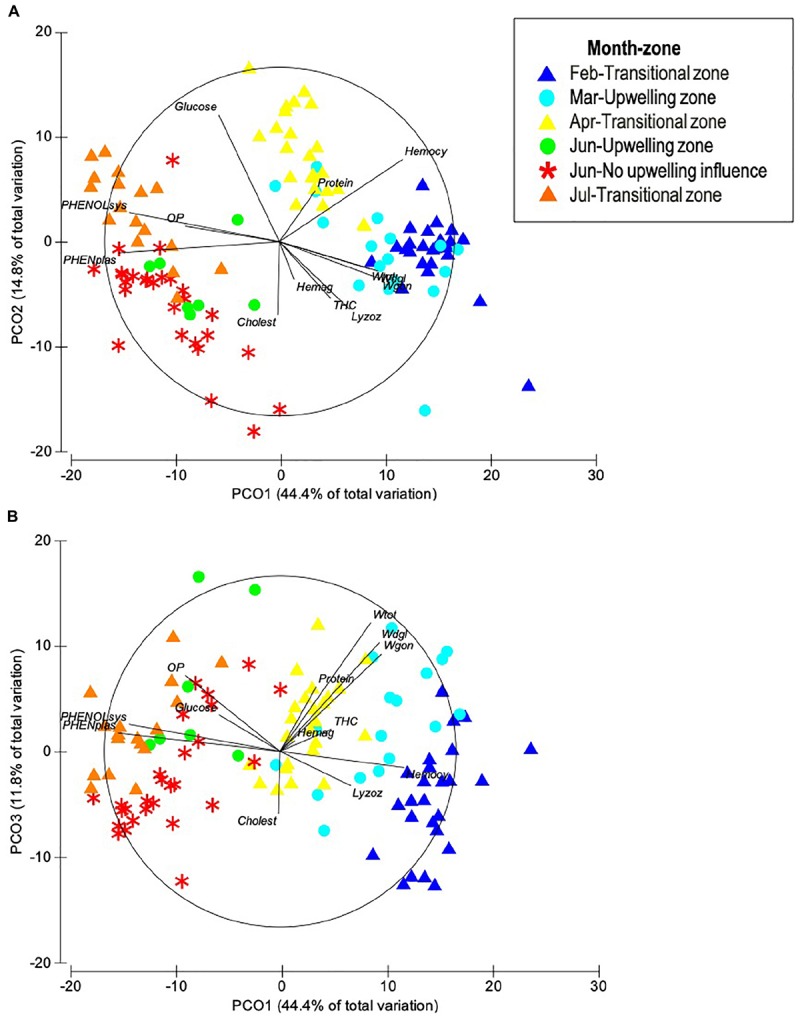
Principal coordinates **(A)** PC1 vs. PC2, and **(B)** PC1 vs. PC3 of eight physiological and five immunological multivariate descriptors measured in adult *O. maya* captured at three zones in the coast of Yucatán on five different months: February, March, and July at the transitional zone (Sisal); March and June at the upwelling zone (Ría Lagartos); and June at the non-upwelling zone (Seybaplaya); *n* = 117.

**FIGURE 2:**
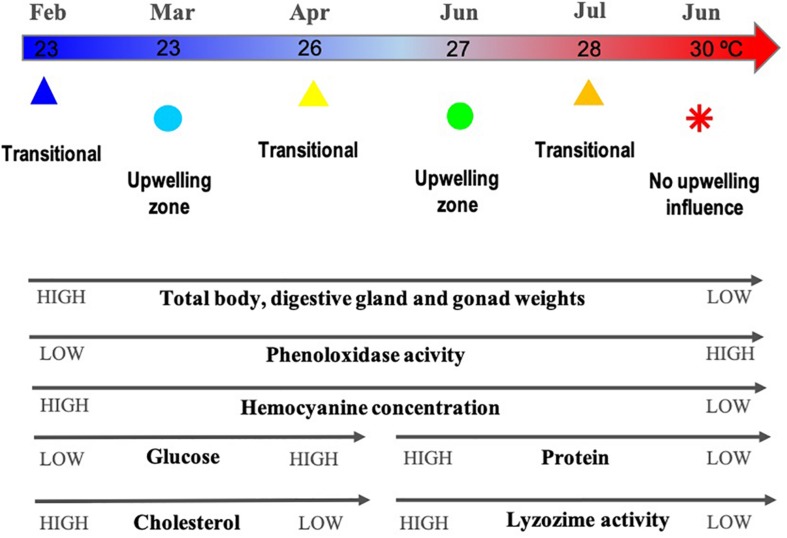
Diagram of variation of the physiological and immunological indicators associated with the thermal gradient related to months/zones where *O. maya* were captured: February, March, and July at the transitional zone (Sisal); March and June at the upwelling zone (Ría Lagartos); and June at the non-upwelling zone (Seybaplaya); *n* = 117. Monthly mean sea surface temperature around the Yucatán Peninsula from National Geophysical Data Center (period 2006–2015) (National Oceanic and Atmospheric Administration [NOAA], 2017).

Ordination by PCoA of the physiological and immunological descriptors of *O. maya* showed that 59.2% of total variation was explained with the first two principal coordinates (Figure 1A) and increased to 71% when a third coordinate was considered (Table 1 and Figure 1B). The PCoA configuration showed how samples representing octopus were effectively ordered in the first and second axes in a way that corresponded to a temperature gradient related to the month – zone in which they were captured (Figure 2). The activity of the phenoloxidase system and total phenoloxidase activity in plasma were strongly correlated with PCoA 1, largely contributing to the separation of samples in the horizontal axis (Table 2). Hemocyanin was also strongly but inversely correlated with the PO system and activity, and to a lesser degree, with the weight of the digestive gland, gonad and total weight. The concentration of glucose and proteins were inversely correlated with cholesterol and lysozyme activity on the vertical axis, so that samples with high glucose and protein concentration were low in cholesterol concentration and lysozyme activity (Table 3). While the third principal coordinate only explained 11.8% of total variation in the data, separation of samples in a third dimension (depth) was mainly given by the digestive gland, gonad, and total weight.

**TABLE 1:** Results of PCoA on eight physiological and five immunological multivariate descriptors measured in adult *O. maya* captured at three locations in the coast of Yucatán on five different months: February, April, and July at transitional zone (Sisal); March and June at the upwelling zone (Ría Lagartos); and June at the non-upwelling zone (Seybaplaya).

	**PCoA 1**	**PCoA 2**	**PCoA 3**
Variation (%)	44.4%	14.8%	11.8%
*Total weight*	0.515	0.155	**-0.734**
*Digestive gland weight*	0.565	0.169	**-0.622**
*Gonad weight*	0.577	0.213	**-0.555**
*Proteins*	0.205	**-0.292**	–0.368
*Cholesterol*	–0.010	**0.420**	0.355
*Glucose*	–0.347	**-0.728**	–0.208
*Osmotic pressure*	**-0.538**	–0.089	–0.432
*Total hemocyte count*	-0.293	0.325	-0.127
*Hemocyanin*	**0.705**	**-0.472**	0.092
*Hemagglutination*	0.085	0.217	–0.060
*Phenoloxidase system*	**-0.860**	–0.168	–0.155
*Phenoloxidase plasma*	**-0.922**	0.064	–0.105
*Lysozyme*	0.402	**0.388**	0.195

*The amount of variation (%) explained by the first three principal coordinates, as well as Spearman correlation coefficients between descriptors and coordinates, are shown. Descriptors contributing the most to the ordination of samples in each principal coordinate are in bold.*

**TABLE 2:** Immunological variables of *Octopus maya* in three zones of Yucatán Peninsula: Seybaplaya, Sisal, and Ría Lagartos.

**Immunological variables**	***N***	**Average**	***SD***
**Total hemocytes count, cel mm^–3^**	**117**	**15,411**	**7,871**
Seybaplaya	27	14,433	8,964
Sisal	66	13,445	5,453
Ría Lagartos	24	21,916	9,013
**Hemagglutination, titer**	**117**	**4.73**	**0.85**
Seybaplaya	27	4.85	0.53
Sisal	66	4.60	0.94
Ría Lagartos	24	4.96	0.81
**Total phenoloxidase plasma OD 490 nm**	**117**	**0.129**	**0.092**
Seybaplaya	27	0.209	0.022
Sisal	66	0.111	0.093
Ría Lagartos	24	0.085	0.086
**Phenoloxidase activity system, OD 490 nm**	**117**	**0.358**	**0.198**
Seybaplaya	27	0.480	0.120
Sisal	66	0.332	0.229
Ría Lagartos	24	0.295	0.099
**Lysozyme, U ml^–1^**	**117**	**8727**	**3951**
Seybaplaya	27	7216	2315
Sisal	66	9091	4632
Ría Lagartos	24	9429	2910

*Number of organisms by locality, average value, standard deviation.*

**TABLE 3:** Physiological variables of *Octopus maya* of three zones of Yucatán Peninsula: Seybaplaya, Sisal, and Ría Lagartos.

**Physiological variables**	***N***	**Average**	***SD***
**Total weight, g**	**117**	**576.13**	**260.72**
Seybaplaya	27	462.49	246.29
Sisal	66	561.43	169.74
Ría Lagartos	24	743.01	384.75
**Digestive gland weight, g**	**117**	**16.14**	**9.45**
Seybaplaya	27	12.28	7.87
Sisal	66	15.53	5.77
Ría Lagartos	24	22.15	15.16
**Hemocyanin, mmol/l**	**117**	**1.45**	**0.62**
Seybaplaya	27	0.88	0.22
Sisal	66	1.72	0.60
Ría Lagartos	24	1.36	0.53
**Osmotic pressure, mOsm kg^–1^**	**117**	**1150**	**27**
Seybaplaya	27	1150	18
Sisal	66	1145	22
Ría Lagartos	24	1164	42
**Proteins, mg ml^–1^**	**117**	**108.73**	**27.39**
Seybaplaya	27	98.01	14.14
Sisal	66	106.48	30.46
Ría Lagartos	24	127.01	20.94
**Cholesterol, mg ml^–1^**	**117**	**0.078**	**0.026**
Seybaplaya	27	0.119	0.011
Sisal	66	0.067	0.012
Ría Lagartos	24	0.062	0.017
**Glucose, mg ml^–1^**	**117**	**0.143**	**0.074**
Seybaplaya	27	0.103	0.026
Sisal	66	0.163	0.084
Ría Lagartos	24	0.129	0.061

*Number of organisms by locality, average value, and standard deviation.*

The multivariate one-way ANOVA showed overall significant differences amongst groups of octopus classified by month – zone of capture (Table 4); paired multivariate *t*-tests showed all six groups were statistically distinguishable from each other (Table 5). Ordination in these axes showed that octopus from February and March collected at the transitional and upwelling zones were among the largest, closely followed by those obtained in April at the transitional zone, and a few of the largest individuals obtained in June at the upwelling zone. Most octopus collected during June and July at the non-upwelling zone and at the transitional zone had intermediate weights. However, some of the individuals in these months and locations were among the smallest collected.

**TABLE 4:** Results of one-way permutational multiple MANOVA’s applied on eight physiological and five immunological multivariate descriptors measured in adult *O. maya* captured at three locations in the coast of Yucatán on five different months: February, April, and July at the transitional zone (Sisal); March and June at the upwelling zone (Ría Lagartos); and June at the non-upwelling zone (Seybaplaya).

**Source of variation**	**df**	**SS**	**MS**	***pseudo-F***	***P***	**Unique permutations**
Month-location	5	21542	4308	38.5	<0.001	9909
Residuals	111	12432	112			
Total	116	33974				

*The degrees of freedom (df), the multivariate sum of squares (SS), mean square (MS), *pseudo-F* and *p*-values, and the number of unique permutations for each test are shown.*

**TABLE 5:** Results of permutational paired *t*-tests that compared centroids representing data of eight physiological and five immunological multivariate descriptors measured in adult *O. maya* captured at three locations in the coast of Yucatán on five different months: February, April, and July at the transitional zone (Sisal); March and June at the upwelling zone (Ría Lagartos); and June at the non-upwelling zone (Seybaplaya).

	**Feb-Sisal**	**Mar-Ría**	**Apr-Sisal**	**Jun-Seyba**	**Jun-Ría**
Mar-Ría	3.50				
Apr-Sisal	6.33	4.24			
Jun-Seyba	8.97	6.37	6.96		
Jun-Ría	6.11	4.02	4.99	3.93	
Jul-Sisal	9.67	6.88	6.69	4.72	3.59

**Pseudo-t* values and permutational *p*-values were all <0.001; between 9803 and 9958 unique permutations were used in tests.*

## Discussion

The physiological and immunological parameters of *O. maya* were studied considering the influence of the seasonal upwelling throughout its geographical distribution in the Yucatán Peninsula, Mexico. Multivariate analyses indicated that the health of the organisms correlated with a temperature gradient, which was in turn linked to the zone and month where the octopuses were captured. The temperature in the studied zones ranged from 22 to 30°C based on sea-surface and from 100 m depth temperatures data from the National Geophysical Data Center (National Oceanic and Atmospheric Administration [NOAA], 2017) and upwelling studies (Zavala-Hidalgo et al., 2006; Enriquez et al., 2013).

It has been demonstrated that high temperatures (28–31°C) affect *O. maya* reproductive capability by inhibiting the spawning of females (Juárez et al., 2015), compromising the maturation of males (López-Galindo et al., 2018), and affecting the viability of eggs and progeny growth (Sanchez-García et al., 2017). In the present study, individuals collected in warmer waters at the non-upwelling zone and in the transitional zone on June and July (28–30°C), both showed high phenoloxidase activity and low hemocyanin concentration. Conversely, individuals captured in the cold months (February, March, and April) in the upwelling and transition zones (Ría Lagartos and Sisal) had low values of both of the aforementioned indicators. The benthic temperature in these two zones was lower (22–27°C) than that of the non-upwelling zone (23–30°C). This data is relevant because to our knowledge this is the first study that evaluate, through immunological and physiological variables, the effect of temperature on health status of wild organisms of *O. maya*.

Immunological adaptation to unfavorable conditions could allow organisms to maintain immunity to avoid infectious diseases caused by opportunistic pathogens. If the exposure to thermal stress is prolonged and/or it happens in combination with a secondary stressor (for example, hypoxia or acidification), this could deteriorate the physiological condition of the individuals, compromising immunity and facilitating disease outbreaks in marine animals (Parisi et al., 2017). Octopuses captured in the warmer waters could be reflecting the metabolic stress associated with temperatures above 27°C, along with immunological compensation mechanisms with higher activity of the phenoloxidase system, despite having a lower concentration of hemocytes, hemocyanin, and proteins than those observed in octopuses captured in upwelling or transition zones (Seybaplaya, Campeche and Sisal, Yucatán), where water temperature is below 26°C, from April to June. To better understand the complex multimeric system that amplifies the immune response and cellular communication in octopuses, the phenoloxidase system was evaluated by the reaction between the humoral components and the elements of degranulated hemocytes in hemolymph, and total phenoloxidase activity in plasma. Low activity of lysozymes in the same organisms could indicate that the higher activity of the phenoloxidase system was not associated with the presence of bacteria in the hemolymph and could be associated to cellular stress, such as oxidative stress. Sanchez-García et al. (2017) investigated the thermal sensitivity of *O. maya* embryos based on growth, respiratory metabolism, and antioxidant mechanisms to define thermal limits. They found that above 27°C embryos experienced negative changes in enzymes involved in antioxidative mechanisms (acetylcholinesterase activity, catalase activities, and total glutathione). Although our work did not include antioxidant metabolism biomarkers, the immunological components analyzed support the hypothesis that immunological compensation occurs when the temperature is above 27°C. However, further experimental work is needed to address such hypothesis.

The fact that octopuses present significant variation in their immunological and physiological condition associated with sea surface temperature opens the possibility of using these organism as bioindicators. Previous monitoring studies have demonstrated that many of the parameters of bivalves can vary significantly among sites and seasons, suggesting that environmental and endogenous factors may affect the immune system and the susceptibility to opportunistic pathogens (Carballal et al., 1998; Duchemin et al., 2007). Hemogram characteristics in a population of cultured mussels revealed that the concentration of circulating *Mytilus galloprovincialis* hemocytes fluctuated following a seasonal pattern (Carballal et al., 1998). Another study of hemocytes from native and invasive Mytilus congeners (*Mytilus californianus* and *Mytilus galloprovincialis*, respectively) found that DNA damage, cellular stress response and apoptosis were induced by acute temperature stress (Yao and Somero, 2012). Paillard et al. (2004) investigated the effect of temperature on the immune response and its relationship with the development of Brown Ring Disease (BRD). Clams kept at different temperatures (8, 14, and 21°C) were experimentally challenged with the pathogen *Vibrio tapetis*, the etiologic agent of BRD. Results demonstrated significant effects of temperature on disease development and on hemolymph immune parameters, including total and viable hemocyte count and, lysozyme activity.

Invertebrate immune reactions are often accompanied by the melanization cascade or proPO activation, which is intimately associated with the onset of factors that stimulate cellular defense by aiding phagocytosis and encapsulation reactions (Sritunyalucksana and Söderhall, 2000). During activation of phenoloxidase system and melanization, some ROS and reactive nitrogen species (RNS) are generated, including superoxide anion (Whitten and Ratcliffe, 1999), nitric oxide (Nappi et al., 2000) and hydrogen peroxide (Dubovskii et al., 2010). These reactive molecules can enhance immunocompetence but uncontrolled can also damage tissues. ROS interaction with the components of the phenoloxidase system could represent an advantage in terms of containment during the melanization process because it takes place at a specific site and for limited time, regulated by antioxidant enzymes such as glutathione which accompany node or capsule formation (see the review, encapsulation and nodulation in insect: Dubovskiy et al., 2016).

Based on the results of the present study, the distribution area of *O. maya* can be divided into two well-differentiated thermal zones: an eastern zone influenced by upwelling pulses and a western zone with no upwelling influence. Studies of reproductive conditions suggest that variations in population parameters could be linked to these thermal zones (Angeles-González et al., 2017). In accordance, a genetic analysis using multilocus microsatellite markers of wild *O. maya* showed that this population is structured in two clusters that match the different thermal zones (Juárez et al., 2018). Whilst the movement of individual *O. maya* throughout the YP during its life cycle has not been determined, direct development and benthic behavior could limit the mobility of the organisms in such a way that health conditions of octopuses might reflect physiological adaptation to regional environmental conditions.

Temperature is one of the main environmental factors that govern the metabolism of marine ectotherms, but the way in which these organisms respond to stress will depend on the thermal tolerance of the species (result of evolutionary processes), and its life history, i.e., the nutritional condition and immunological adaptation could also be crucial to their ability to absorb the effects of thermal stress. For example, Parisi et al. (2017), studied the combined effects of changes in temperature and food availability plus increased hypoxia in the marine mussel *Mytilus galloprovincialis*. They found that an increase in temperature affected the functionality of esterase and alkaline phosphatase enzymes. However, under normoxic conditions, food had a buffering effect that counteracted the negative effects of high temperature. At extreme temperatures, energy demands of metabolism might not be satisfied because organisms are unable to supply enough oxygen at the cell level (Pörtner and Farrel, 2008). This hypothesis further states that the aerobic scope, growth, activity, maintenance, reproduction, and storage are linked to the physiological responses which depend on ATP produced by aerobic metabolism (Sokolova et al., 2012). Hence, physiological and immunological markers can be useful to understand the health condition of organisms by reflecting the metabolic adjustments linked to energy demands and defense mechanisms in extreme conditions.

Stress is a general adaptive reaction crucial for survival and basically positive involving the neuroendocrine and the immune systems. Studies on biological and evolutionary implications of stress response indicate that in all bilaterian metazoans, the molecular mediators of the stress response, i.e., corticotrophin-releasing hormone, corticotrophin, catecholamines, and glucocorticoids, have been preserved during evolution (Ottaviani and Malagoli, 2009; Heather et al., 2015). The neuroendocrine and immune response shows a combinatorial strategy where the repetitive use of a set of signaling molecules is shared by the immune and neuroendocrine systems for different functions (Di Cosmo and Polese, 2016). A clear example of this intricate correlation is observed during the eggs-caring period: female octopuses spawn once in their life and eat less or stop feeding when caring for the eggs (Wodinsky, 1977). Research on the health status of *O. maya* females on different days after spawning reflected consumption of reserves coinciding with an increased immune process characterized by hemagglutination and phenoloxidase activity (Roumbedakis et al., 2017). These results show that despite the long starvation period, females sustain an adequate state of health to care for their spawn by resorting to immune compensation, in the opposite direction to the energy metabolism of the organisms.

Most information on the immune system and physiological condition of cephalopods comes from research carried out under laboratory conditions (Hanlon and Messenger, 1996; Gestal and Castellanos-Martínez, 2015; Roumbedakis et al., 2017). Although field observations are difficult to perform and involve the interaction of diverse environmental factors that affect the physio-immunological condition of the organisms, they can contribute with valuable information to better understand the connection between environment and organismal immunity. This information could be relevant to make predictions about the effects of climate change and environment on immunocompetence and disease outbreaks.

## Conclusion

In conclusion, temperature is a key factor that modulates *O. maya’s* reproduction, early development, metabolism, and immune system. Results obtained from wild octopuses show that organisms from natural temperatures lower than 27°C (upwelling zone and transitional zone in March and April) are in better conditions indicated by larger size, high concentrations of hemocyanin and low activity of the phenoloxidase system. Our work complements the studies carried out on the species’ thermal tolerance, reinforcing the idea that *O. maya* sensitivity to temperature may be relevant in monitoring programs to detect environmental changes associated with global warming.

## Ethics Statement

The Mexican official norm (NOM-062-ZOO-1999) on the technical specifications for the production, use and care of laboratory animals does not include fish or invertebrates, and regulations on the matter are scarce. We followed the Guide for the Care and Use of Experimental Animals in Research and Teaching of the Faculty of Superior Studies-Cuautitlán (http://www.cuautitlan.unam.mx/) at Universidad Nacional Autónoma de México.

## Author Contributions

CP, CR, HC-L, AS, and RR-C designed and ran laboratory analysis. MM and CP analyzed and interpreted the data. CP, RR-C, MM, AS, HC-L, and PG contributed to writing the manuscript. All authors reviewed, edited and approved the final manuscript.

## Conflict of Interest Statement

The authors declare that the research was conducted in the absence of any commercial or financial relationships that could be construed as a potential conflict of interest.

## References

[B1] Aguila J. Cuzon G. Pascual C. Domingues P. M. Gaxiola G. Sánchez A. (2007). The effects of fish hydrolysate (CPSP) level on *Octopus maya* (Voss and Solis) diet: digestive enzyme activity, blood metabolites, and energy balance. *Aquaculture* 273 641–655. 10.1016/j.aquaculture.2007.07.010

[B2] Anderson M. J. (2001). A new method for non-parametric multivariate analysis of variance. *Austral Ecol.* 26 32–46. 10.1111/j.1442-9993.2001.01070.pp.x

[B3] Angeles-González L. E. Calva R. Santos -Valenzia J. Avila- Poveda O. Olivares A. Diaz F. (2017). Temperature modulates spatio-temporal variability of the functional reproductive maturation of *Octopus maya* (Cephalopoda) on the shelf of the yucatan peninsula, Mexico. *J. Molluscan Stud.* 83 280–288. 10.1093/mollus/eyx013

[B4] Beuerlein K. Löhr S. Westermann B. Ruth P. Schipp R. (2002). Components of the cellular defense and detoxification system of the common cuttlefish *Sepia officinalis* (Mollusca, Cephalopoda). *Tissue Cell* 34 390–396. 10.1016/S0040816602000708 12441091

[B5] Boyle P. R. (1976). Receptor units responding to movement in the octopus mantle. *J. Exp. Biol.* 65 1–9. 993696 10.1242/jeb.65.1.1

[B6] Bradford M. M. (1976). A rapid and sensitive method for the quantitation of microgram quantities of protein utilizing the principle of protein-dye binding. *Anal. Biochem.* 72 248–254. 10.1016/0003-2697(76)90527-3 942051

[B7] Caamal-Monsreal C. Mascaró M. Gallardo P. Rodríguez S. Noreña-Barroso E. Domingues P. (2015). Effects of maternal diet on reproductive performance of *O. maya* and its consequences on biochemical characteristics of the yolk, morphology of embryos and hatchling quality. *Aquaculture* 441 84–94. 10.1016/J.AQUACULTURE.2015.01.020

[B8] Caamal-Monsreal C. Uriarte I. Farias A. Díaz F. Sánchez A. Re D. (2016). Effects of temperature on embryo development and metabolism of *O. maya*. *Aquaculture* 451 156–162. 10.1016/J.AQUACULTURE.2015.09.011

[B9] Campa-Córdova A. I. Hernández-Saavedra N. Y. De Philippis R. Ascencio F. (2002). Generation of superoxide anion and SOD activity in haemocytes and muscle of American white shrimp (*Litopenaeus vannamei*) as a response to β-glucan and sulphated polysaccharide. *Fish Shellfish Immunol.* 12 353–366. 10.1006/FSIM.2001.0377 12049170

[B10] Carballal M. J. Lopez C. Azevedo C. Villalba A. (1998). Hemolympe cell types of mussel *Mytilus galloprovincialis*. *Dis. Aquat. Organ.* 29 127–135. 10.3354/dao029127

[B11] Chen C. Cheng S. Y. (1993a). Hemolymph PCO2, hemocyanin, protein level and urea excretions of *Penaeus monodon* exposed to ambient ammonia. *Aquat. Toxicol.* 27 281–292.

[B12] Chen C. Cheng S. Y. (1993b). Studies on hemocyanin and hemolymph protein levels of *Penaeus japonicus* based on sex, size, and moulting cycle. *Comp. Biochem. Physiol. B* 106 293–296.

[B13] Cruz-López H. (2010). *Caracterización Estacional de la Condición Fisiológica de la Población Silvestre del Pulpo Rojo Octopus Maya (Voss y Solís-Ramírez, 1966) en la Localidad de Sisal, Yucatán, México.* Guadalajara: UAG.

[B14] Di Cosmo A. Polese G. (2016). Neuroendocrine-immune systems response to environmental stressors in the cephalopod *Octopus vulgaris*. *Front. Physiol.* 7:434. 10.3389/fphys.2016.00434 27733834 PMC5039199

[B15] Dubovskii I. Grizanova E. Chertkova E. Slepneva I. Komarov D. Vorontsova Y. (2010). Generation of reactive oxygen species and activity of antioxidants in hemolymph of the moth larvae *Galleria mellonella* (L.) (Lepidoptera: Piralidae) at development of the process of encapsulation. *J. Evol. Biochem. Physiol.* 46 35–43. 20297667

[B16] Dubovskiy I. Kryukova N. Glupov V. Ratcliffe N. (2016). Encapsulation and nodulation in insect. *Invertebrate Surviv. J.* 13 229–246.

[B17] Duchemin M. Wessel N. Fournier M. Auffret M. (2007). Flow cytometric measurement of the clearance rate in the blue mussel *Mytilus edulis* and the development of a new individual exposure system for aquatic immunotoxicological studies. *Environ. Pollut.* 153 492–496. 17905494 10.1016/j.envpol.2007.08.017

[B18] Enriquez C. Mariño-Tapia I. Jeronimo G. Capurro-Filograsso L. (2013). Thermohaline processes in a tropical coastal zone. *Cont. Shelf Res.* 69 101–109. 10.1016/j.csr.2013.08.018

[B19] Fazio F. (2019). Fish hematology analysis as an important tool of aquaculture: a review. *Aquaculture* 500 237–242.

[B20] Fiorito G. Affuso A. Basil J. Cole A. Girolamo P. D’Angelo L. (2015). guidelines for the care and welfare of cephalopods in research – a consensus based on an initiative by CephRes, FELASA and the boyd group. *Lab. Anim.* 49 1–90. 10.1177/0023677215580006 26354955

[B21] Fisher W. S. DiNuzzo A. R. (1991). Agglutination of bacteria and erythrocytes by serum from six species of marine molluscs. *J. Invertebr. Pathol.* 57 380–394. 10.1016/0022-2011(91)90142-D 2066577

[B22] Gallardo P. Olivares A. Martínez-Yáñez R. Caamal-Monsreal C. Domingues P. M. Mascaró M. (2017). Digestive Physiology of *Octopus maya* and *O. mimus*: temporality of Digestion and Assimilation Processes. *Front. Physiol.* 8:355. 10.3389/fphys.2017.00355 28620313 PMC5450419

[B23] Gestal C. Castellanos-Martínez S. (2015). Understanding the cephalopod immune system based on functional and molecular evidence. *Fish Shellfish Immunol.* 46 120–130. 10.1016/j.fsi.2015.05.005 25982402

[B24] Hanlon R. T. Forsythe J. W. (1985). Advances in the laboratory culture of octopuses for biomedical research. *Lab. Anim. Sci.* 35 33–40. 3981958

[B25] Hanlon R. T. Messenger J. B. (1996). *Cephalopod Behaviour.* Cambridge: Cambridge University Press.

[B26] Heather D. Taewoo R. Donelson J. Van Herwerden L. Seridi L. Ghosheh Y. (2015). Molecular processes of transgenerational acclimation to a warming ocean. *Nat. Clim. Chang.* 5 1074–1078.

[B27] Hernández-López J. Gollas-Galván T. Vargas-Albores F. (1996). Activation of the prophenoloxidase system of the brown shrimp (*Penaeus californiensis* Holmes). *Comp. Biochem. Physiol. C Pharmacol. Toxicol. Endocrinol.* 113 61–66. 10.1016/0742-8413(95)02033-0

[B28] Hochner B. Shomrat T. Fiorito G. (2006). The octopus: a model for a comparative analysis of the evolution of learning and memory mechanisms. *Biol. Bull.* 10 308–317. 10.2307/4134567 16801504

[B29] Juárez O. E. Enríquez L. Camarena-Rosales F. Arena L. Galindo-Sánchez C. E. Lafarga-De la Cruz F. (2018). Genetic monitoring of the mexican four-eyed octopus *Octopus maya* population: new insights and perspectives for the fishery management. *Fish. Res.* 206 109–114. 10.1016/J.FISHRES.2018.05.002

[B30] Juárez O. E. Galindo-Sánchez C. E. Díaz F. Re D. Sánchez-García A. M. Camaal-Monsreal C. (2015). Is temperature conditioning *Octopus maya* fitness? *J. Exp. Mar. Bio. Ecol.* 467 71–76. 10.1016/j.jembe.2015.02.020

[B31] Legendre P. Legendre L. (1998). *Numerical Ecology.* 2nd Edn Amsterdam: Elsevier, 853.

[B32] Lignot J.-H. Spanings-Pierrot C. Charmantier G. (2000). Osmoregulatory capacity as a tool in monitoring the physiological condition and the effect of stress in crustaceans. *Aquaculture* 191 209–245. 10.1016/S0044-8486(00)00429-4

[B33] Linares M. Caamal-Monsreal C. Olivares A. Sánchez A. Rodríguez S. Zúñiga O. (2015). Timing of digestion, absorption and assimilation in octopus species from tropical (*Octopus maya*) and subtropical-temperate (*O. mimus*) ecosystems. *Aquat. Biol.* 24 127–140. 10.3354/ab00642

[B34] Locatello L. Fiorito G. Finos L. Rasotto M. B. (2013). Behavioural and immunological responses to an immune challenge in *Octopus vulgaris*. *Physiol. Behav.* 122 93–99. 10.1016/J.PHYSBEH.2013.08.029 24021926

[B35] López-Galindo L. Galindo-Sánchez C. Olivares A. Avila-Poveda O. H. Díaz F. Juárez O. E. (2018). Reproductive performance of *Octopus maya* males conditioned by thermal stress. *Ecol. Indic.* 96 437–447. 10.1016/J.ECOLIND.2018.09.036

[B36] Markaida U. Méndez-Loeza I. Rosales-Raya M. L. (2017). ). Seasonal and spatial trends of mayan octopus, *Octopus maya*, population dynamics from campeche, Mexico. *J. Mar. Biol. Assoc. U.K.* 97 1663–1673. 10.1017/S0025315416001132

[B37] Martínez R. Gallardo P. Pascual C. Navarro J. C. Sánchez A. Caamal-Monsreal C. (2014). Growth, survival and physiological condition of *Octopus maya* when fed a successful formulated diet. *Aquaculture* 42 310–317.

[B38] McArdle B. H. Anderson M. J. (2001). Fitting multivariate models to community data: a comment on distance-based redundancy analysis. *Ecology* 82 290–297.

[B39] Merino M. (1997). Upwelling on the yucatan shelf: hydrographic evidence. *J. Mar. Syst.* 13 101–121. 10.1016/S0924-7963(96)00123-6

[B40] Moguel C. Mascaró M. Avila-Poveda O. Caamal-Monsreal C. Sánchez A. Pascual C. (2010). Morphological, physiological, and behavioural changes during post-hatching development of *Octopus maya* (Mollusca: Cephalopoda) with special focus on digestive system. *Aquat. Biol.* 9 35–48.

[B41] Muñoz M. Cedeño R. Rodríguez J. Van Der Knaap W. P. W. Mialhe E. Bachère E. (2000). Measurement of reactive oxygen intermediate production in haemocytes of the penaeid shrimp, *Penaeus vannamei*. *Aquaculture* 191 89–107. 10.1016/S0044-8486(00)00420-8

[B42] Mydlarz L. D. Jones L. E. Harvell C. D. (2006). Innate immunity, environmental drivers, and disease ecology of marine and freshwater invertebrates. *Annu. Rev. Ecol. Evol. Syst.* 37 251–288. 10.1146/annurev.ecolsys.37.091305.110103

[B43] Nappi A. J. Vass E. Frey F. Carton Y. (2000). Nitric oxide involvement in *Drosophila* immunity. *Nitric Oxide* 4 423–430. 10944427 10.1006/niox.2000.0294

[B44] National Oceanic and Atmospheric Administration [NOAA] (2017). *State of the Climate: Global Climate Report for Annual 2016.* Available at: https://www.ncdc.noaa.gov/sotc/global/201613

[B45] Noyola J. Caamal-Monsreal C. Díaz F. Re D. Sánchez A. Rosas C. (2013a). Thermopreference, tolerance and metabolic rate of early stages juvenile *Octopus maya* acclimated to different temperatures. *J. Therm. Biol.* 38 14–19. 10.1016/J.JTHERBIO.2012.09.001 24229799

[B46] Noyola J. Mascaró M. Caamal-Monsreal C. Noreña-Barroso E. Díaz F. Re D. (2013b). Effect of temperature on energetic balance and fatty acid composition of early juveniles of *Octopus maya*. *J. Exp. Mar. Bio. Ecol.* 445 156–165. 10.1016/J.JEMBE.2013.04.008

[B47] Ottaviani E. Malagoli D. (2009). Around the word stress: its biological and evolutive implications. *Invertebrate Surviv. J.* 6 1–6.

[B48] Paillard C. Le Roux F. Borrego J. J. (2004). Bacterial disease in marine bivalves, a review of recent studies: trends and evolution. *Aquat. Living Resour.* 17 477–498. 10.1051/alr:2004054

[B49] Parisi M. Mauro M. Sara G. Cammarata M. (2017). Temperature increases, hypoxia, and changes in food availability affect immunological biomarkers in the marine mussel *Mytilus galloprovincialis*. *J. Comp. Physiol. B* 187 1117–1126. 10.1007/s00360-017-1089-2 28389696

[B50] Parry R. M. Chandan R. C. Shahani K. M. (1965). A rapid and sensitive assay of muramidase. *Exp. Biol. Med.* 119 384–386. 10.3181/00379727-119-30188 14328897

[B51] Pascual C. Arena L. Cuzon G. Gaxiola G. Taboada G. Valenzuela M. (2004). Effect of a size-based selection program on blood metabolites and immune response of *Litopenaeus vannamei* juveniles fed different dietary carbohydrate levels. *Aquaculture* 230 405–416. 10.1016/S0044-8486(03)00438-1

[B52] Pascual C. Sánchez A. Sánchez A. Vargas-Albores F. LeMoullac G. Rosas C. (2003). Haemolymph metabolic variables and immune response in *Litopenaeus setiferus* adult males: the effect of an extreme temperature. *Aquaculture* 218 637–650. 10.1016/S0044-8486(02)00300-9

[B53] Pascual-Jiménez C. Huchin-Mian J. P. Simões N. Briones-Fourzán P. Lozano-Álvarez E. Sánchez-Arteaga A. (2012). Physiological and immunological characterization of Caribbean spiny lobsters *Panulirus argus* naturally infected with *Panulirus argus* virus 1 (PaV1). *Dis. Aquat. Organ.* 100 113–124. 10.3354/dao02497 23186699

[B54] Pörtner H. O. Farrel A. (2008). Physiology and climate change. *Science* 322 690–692. 10.1126/science.1163156 18974339

[B55] Rögener W. Renwrantz L. Uhlenbruck G. (1987). Analysis of *Octopus vulgaris* hemolymph containing a glycoprotein with blood group A-like properties. *Comp. Biochem. Physiol. B Biochem.* 86 347–351. 10.1016/0305-0491(87)90304-X

[B56] Roper C. F. E. Sweeney M. J. Nauen C. E. (1984). Cephalopods of the world: an annotated and illustrated catalogue of species of interest to fisheries. *FAO Fish. Synop.* 125 1–277.

[B57] Rosas C. Cuzon G. Gaxiola G. Pascual C. Taboada G. Arena L. (2002). An energetic and conceptual model of the physiological role of dietary carbohydrates and salinity on *Litopenaeus vannamei* juveniles. *J. Exp. Mar. Biol. Ecol.* 268 47–67.

[B58] Roumbedakis K. Mascaró M. Martins M. L. Gallardo P. Rosas C. Pascual C. (2017). Health status of post-spawning *Octopus maya* (Cephalopoda: Octopodidae) females from Yucatan Peninsula, Mexico. *Hydrobiologia* 808 23–34. 10.1007/s10750-017-3340-y

[B59] Sánchez A. Pascual C. Saìnchez A. Vargas-Albores F. Le Moullac G. Rosas C. (2001). Hemolymph metabolic variables y immune response in *Litopenaeus setiferus* adult males: the effect of acclimation. *Aquaculture* 198 13–28.

[B60] Sanchez-García A. Rodríguez-Fuentes G. Díaz F. Galindo-Sánchez C. E. Ortega K. Mascaró M. (2017). Thermal sensitivity of *Octopus maya* embryos as a tool for monitoring the effects of environmental warming in the Southern of Gulf of Mexico. *Ecol. Indic.* 72 574–585. 10.1016/J.ECOLIND.2016.08.043

[B61] Sieiro M. P. Aubourg S. P. Rocha F. (2006). Seasonal study of the lipid composition in different tissues of the common octopus (*Octopus vulgaris*). *Eur. J. Lipid Sci. Technol.* 108 479–487. 10.1002/ejlt.200500322

[B62] Söderhäll K. Häll L. (1984). Lipopolysaccharide-induced activation of prophenoloxidase activating system in crayfish haemocyte lysate. *BBA Gen. Subj.* 797 99–104. 10.1016/0304-4165(84)90387-8

[B63] Söderhäll K. Smith V. J. (1983). Separation of the haemocyte populations of Carcinus maenas and other marine decapods, and prophenoloxidase distribution. *Dev. Comp. Immunol.* 7 229–239. 10.1016/0145-305X(83)90004-6 6409683

[B64] Sokolova I. M. Frederich M. Bagwe R. Lannig G. Sukhotin A. A. (2012). Energy homeostasis as an integrative tool for assesing limits of environmental stress tolerance in aquatic invertebrates. *Mar. Environ. Res.* 79 1–15. 10.1016/j.marenvres.2012.04.003 22622075

[B65] Song Y. L. Hsieh Y. T. (1994). Immunostimulation of tiger shrimp (*Penaeus monodon)* hemocytes for generation of microbicidal substances: analysis of reactive oxygen species. *Dev. Comp. Immunol.* 18 201–209. 8001699 10.1016/0145-305x(94)90012-4

[B66] Sritunyalucksana K. Söderhall K. (2000). The proPO and clotting system in crustaceans. *Aquaculture* 191 53–69.

[B67] Sung H. H. Chang H. J. Her C. H. Chang J. C. Song Y. L. (1998). Phenoloxidase Activity of Hemocytes Derived from *Penaeus monodonand* and *Macrobrachium rosenbergii*. *J. Invertebr. Pathol.* 71 26–33. 10.1006/jipa.1997.4703 9446734

[B68] Tercero J. F. Rosas C. Mascaro M. Poot G. Domingues P. Noreña E. (2015). Effects of parental diets supplemented with different lipid sources on *Octopus maya* embryo and hatching quality. *Aquaculture* 448 234–242. 10.1016/J.AQUACULTURE.2015.05.023

[B69] Vargas-Albores F. Jiménez-Vega F. Söderhäll K. (1996). A plasma protein isolated from brown shrimp (*Penaeus californiensis*) which enhances the activation of prophenoloxidase system by β-1,3-glucan. *Dev. Comp. Immunol.* 20 299–306. 10.1016/S0145-305X(96)00007-9 9016384

[B70] Vargas-Albores F. Jiménez-Vega F. Yepiz-Plascencia G. M. (1997). Purification and comparison of beta-1,3-glucan binding protein from white shrimp (*Penaeus vannamei*). *Comp. Biochem. Physiol. B. Biochem. Mol. Biol.* 116 453–458. 9149399 10.1016/s0305-0491(96)00268-4

[B71] Voss G. L. Solís-Ramírez M. J. (1966). *Octopus maya*, a new species from the bay of campeche. *Bull. Mar. Sci.* 16 615–625.

[B72] Whitten M. M. A. Ratcliffe N. A. (1999). In vitro superoxide activity in the haemolymph of the West Indian leaf cockroach, *Blaberus discoidalis*. *J. Insect Physiol.* 45 667–675. 12770352 10.1016/s0022-1910(99)00039-6

[B73] Wodinsky J. (1977). Hormonal inhibition of feeding and death in octopus-control by optic gland secretion. *Science* 198 948–951. 17787564 10.1126/science.198.4320.948

[B74] Yao C. L. Somero G. N. (2012). The impact of acute temperature stress on hemocytes of invasive and native mussels (*Mytilus galloprovincialis* and *Mytilus californianus*): DNA damage, membrane integrity, apoptosis and signaling pathways. *J. Exp. Biol.* 215(Pt 24), 4267–4277. 10.1242/jeb.073577 22972891

[B75] Zavala-Hidalgo J. Gallegos-García A. Martínez-López B. Morey S. L. O’Brien J. J. (2006). Seasonal upwelling on the western and southern shelves of the gulf of Mexico. *Ocean Dyn.* 56:333 10.1007/s10236-006-0072-3

